# Assessing the swelling behavior of oil paint in fatty acid methyl esters (FAMEs)†

**DOI:** 10.1039/d4ra07464e

**Published:** 2024-12-17

**Authors:** Chiara Biribicchi, Michael Doutre, Gabriele Favero

**Affiliations:** a Department of Earth Sciences, Sapienza University of Rome P.le Aldo Moro 5 Rome 00185 Italy chiara.biribicchi@uniroma1.it; b UCLA/Getty Interdepartmental Program in the Conservation of Cultural Heritage, University of California A210 Fowler Building/Box 951510, 308 Charles E. Young Dr North Los Angeles CA 90095 USA; c Getty Conservation Institute (GCI) 1200 Getty Center Drive, Suite 700 Los Angeles CA 90049 USA michael.doutre@pc.gc.ca; d Department of Environmental Biology, Sapienza University of Rome Piazzale Aldo Moro 5 Rome 00185 Italy gabriele.favero@uniroma1.it

## Abstract

Fatty Acid Methyl Esters (FAMEs) have gained attention as low-impact solvents, offering low toxicity and versatility in applications ranging from biofuels to chemical feedstocks. This study investigates the swelling behavior of naturally-aged zinc white oil paint fragments when exposed to FAMEs with varying chain lengths. Swelling was monitored using a microscopy-based image analysis technique over a 15 minutes period, demonstrating that FAMEs induce low to moderate swelling in oil paints, consistent with prior findings on solvent–paint interactions. The degree of swelling is influenced by the molecular properties of the solvents, such as polarity, chain length, and rate of diffusion. Additionally, the study underscores the importance of considering the competing processes of swelling and leaching, where solvent penetration can lead to the extraction of low-molecular-weight components from the paint matrix. This research underscores the need for careful solvent selection in conservation practices to minimize the risk of swelling-induced damage and leaching. Further studies are required to fully understand the long-term effects of FAMEs on oil paint stability and integrity.

## Abstract 2

Naturally aged zinc white oil paint exhibits low-to-moderate swelling in FAMEs. Swelling differences are influenced by the chain length of the FAMEs.

## Graphical abstract

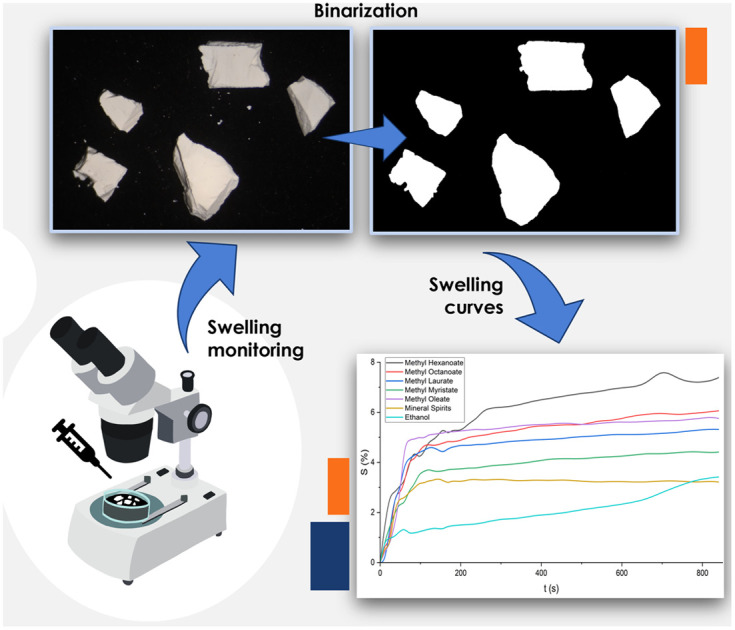

## Introduction

Fatty Acid Methyl Esters (FAMEs) have been studied as a bio-based and low-toxic fuel alternative to petroleum diesel. Beyond fuel, FAMEs demonstrate versatility, finding utility as solvents, lubricants, and chemical feedstocks.^1,2^ FAMEs, produced through the transesterification of triglycerides from renewable and recyclable sources like vegetable oils or crude materials, have gained attention for their wide range of applications.^3–6^ Their advantages, including biodegradability, renewability, and reduced dependence on limited resources, contribute to their lower environmental impact.^3^ Their solubility parameters and eco-friendly attributes make FAMEs a promising alternative to conventional hazardous low-polar solvents such as petroleum derived aliphatic hydrocarbons.^7,8^ However, their potential interaction with the original materials of artworks needs to be thoroughly investigated before they can be utilized in conservation practice.

In oil paintings, a significant risk to the integrity of the original paint layer arises from the phenomenon of swelling, which occurs due to the absorption of cleaning solvents. This swelling leads to a more open and flexible paint structure, thereby increasing its susceptibility to pigment loss when exposed to mechanical action, such as the application of a swab during varnish removal. Consequently, the swollen paint becomes more vulnerable to damage, as the physical interaction with the cleaning swab can dislodge pigments and compromise the artwork's original texture and appearance.^9–12^

Swelling induces both physical and chemical changes within a paint film, generating internal stresses as the film expands and softens.^9^ This softening enhances the paint's susceptibility to absorbing dirt, pollutants, and facilitates the movement of extractable components within the layer.^9,10^ The swelling phenomenon predominantly involves the interaction between the solvent and the polymeric structure of the binding medium. Despite numerous efforts to correlate swelling behavior with a single solvent parameter, these attempts have consistently yielded incomplete results which do not take into account other factors, such as the specific features of the polymer.^13–16^ In reality, the swelling response and solvent sensitivity are influenced by a complex interplay of factors, including solvent polarity and molecular structure, the degree of crosslinking within the polymer matrix, the concentration of plasticizers, environmental conditions experienced over the painting's lifetime, and the presence of ions.^17^ Given the diversity of polymeric structures in paint materials, the interactions between polymers and solvents vary significantly.

Although pigments and colorants do not directly impact the swelling behavior of a paint film, certain inorganic pigments can affect the drying process of oils by releasing metal ions, which in turn contribute to the degradation of the binding medium. This can indirectly influence the polymer properties of the binder. Additionally, some pigments exhibit inherent solubility in organic solvents, particularly in the case of organic pigments.^18–20^

The phenomenon of swelling is closely tied to mass transport – specifically the diffusion of solvents into the paint matrix – as well as the thermodynamics, and solvent evaporation and retention. The rates of swelling and deswelling are largely governed by the solvent's diffusion within the paint film and its evaporation from the surface.^17^

In the 1950s, Stolow conducted pioneering studies aimed at predicting paint swelling based on solvent properties, laying the groundwork for the use of Hansen and Teas solubility parameters in this context.^15,21–23^ Stolow's concept of a “peak swelling region” for oil paint was later incorporated into the Teas chart to aid conservators in selecting solvents outside this region.^14^ Although these models continue to guide solvent selection, subsequent research has highlighted their limitations.^24–26^ The swelling behavior of paint is highly dependent on factors such as paint type and age, and may extend beyond the defined “peak swelling region” when considering lower degrees of swelling. This creates multiple swelling regions, complicating the correlation between solvent solubility parameters and the swelling peak.^26,27^

Moreover, older paint films exhibit different swelling behaviors compared to the younger films used in Stolow's studies, due to higher concentrations of oxidized functional groups, which alter solubility parameters and the composition of extractable components.^27^ Studies comparing young and aged paint films have shown that younger films contain relatively higher levels of oxidized C_18_ fatty acids and lower levels of diacids, reflecting active oxidation processes in the paint.^28^ This complexity further outlines the multifactorial nature of swelling, which is influenced by polymer properties, paint composition, aging processes, and lifetime, and is interconnected with other phenomena occurring during solvent–paint interactions.^16^

One such phenomenon is leaching, which invariably accompanies solvent diffusion into the paint layer, even though it cannot be noticed by the naked eye.^28–34^ Various studies have detected the removal of soluble fatty acids – primarily palmitic and stearic acids – from the paint through swabbing with solvents, as confirmed *via* gas chromatography-mass spectrometry (GC-MS), demonstrating that leaching can occur in real paintings, even with brief solvent contact times.^29,31,34^

Leaching in oil paints involves the migration of low-molecular-weight molecules (LMWMs) to the surface as the solvent penetrates the bulk of the paint. This process is influenced by the condition of the paint, including its age, the presence of cracks, and the availability of soluble or extractable molecules.^17^ Leaching can increase the density of the paint, produce surface haze by redistributing or removing small molecules, induce chemical degradation, and cause embrittlement by extracting LMWMs that act as plasticizers within the polymer matrix.^16,29,35–43^ Swelling and leaching are therefore interconnected; faster solvent diffusion leads to more rapid swelling, leaching, and evaporation.^44^ At the same time, these processes can compete with each other, as the swelling-induced expansion of the paint can be counteracted by the contraction caused by the loss of binder materials. Consequently, high-leaching solvents may appear to induce less swelling due to the opposing effects of expansion and contraction.^11^

Due to the limited research on the swelling behavior of oil paints in fatty acid methyl esters (FAMEs) with varying chain lengths – and consequently differing solubility parameters and physicochemical properties, despite belonging to the same solvent class – an in-plane swelling analysis was performed using a microscopy-based image analysis method, as outlined by Phenix (2002).^11^ For this study, zinc oxide-based paint fragments from a 1966 oil on canvas painting were used. This choice was made because oil paint binding media containing zinc or lead-based pigments have been reported to exhibit high swelling behavior and closely resemble metal-ion-containing polymers, known as ionomers, which have recently been recognized as suitable models for mature oil paint binders.^45–47^

## Materials and methods

### Selection of the greener formulations

To investigate the dissolution ability of Fatty Acid Methyl Esters (FAMEs) and compare their behavior based on alkyl chain length, five FAMEs with chain lengths ranging from C_6_ to C_18_ were selected, taking into account their physicochemical and toxicological properties (Table 1). The solubility parameters of these FAMEs were evaluated using Hansen Solubility Parameters (HSPs), with the center of the HSP sphere representing the solvent most commonly used for removing such substances from artifacts, namely, mineral spirits (MS).^15,48^ These HSPs were further translated into Teas Fractional Parameters.^23^ In terms of toxicological considerations, FAMEs known to be harmful or lacking comprehensive toxicological data were excluded. The selection process was guided by hazard classifications provided by the European Chemicals Agency (ECHA).^49^

**Table 1:** Hansen Solubility parameters (*δ*_D_, *δ*_P_, *δ*_H_) with RED value; Teas fractional parameters (*F*_d_, *F*_p_, *F*_h_); boiling point (BP), and vapor pressure (*P*°)

Compound	*δ*_D_	*δ*_P_	*δ*_H_	RED	*F*_d_	*F*_p_	*F*_h_	BP (°C)	*P*° (kPa@25 °C)
Mineral spirits (MS)	15.8 (ref. 50)	0.1 (ref. 50)	0.2 (ref. 50)	0.00	100.00	0.00	0.00	98	2.7
Methyl hexanoate (MH)	16	4.3	5.8	1.00	61.30	16.48	22.22	149.5	0.50
Methyl octanoate (MO)	15.4	2.7	5.9	0.90	64.17	11.25	24.58	192.6	0.07
Methyl laurate (ML)	16	2.1	5.2	0.77	68.67	9.01	22.32	262	5.5 × 10^−4^
Methyl myristate (MM)	16	1.9	4.2	0.63	72.40	8.60	19.00	295.9	6.5 × 10^−5^
Methyl oleate (MOL)	16.1	1.5	3.5	0.52	76.30	7.11	16.59	351	8.4 × 10^−7^

### Characterization of the oil paint

The zinc white-based paint used for the experiment was characterized using Pyrolysis-Gas Chromatography/Mass Spectrometry (Py-GC/MS) and Fourier Transform Infrared Spectroscopy (FT-IR) in attenuated total reflectance mode (ATR) to obtain information about its components and relate them to the observed swelling.

An Agilent thermal separation probe was used for pyrolysis. 2.5 µmL of 5% (v/v) TMAH in MeOH was added to the pyrolysis probe to methylate the sample. The pyrolysis interface was ramped from 50–450 °C at 900 °C min^−1^ and held for 3 minutes. The pyrolyzer was interfaced to an Agilent Technologies 5975C inert MSD/7890A gas chromatograph/mass spectrometer. A J&W DB-5ms Ultra Inert GC Column, 30 m, 0.25 mm, 0.25 µm capillary column was used for the separation with helium carrier gas set to 1.2 ml min^−1^. The split injector was set to 320 °C with a split ratio of 1 : 25 and a 3 minutes solvent delay. The GC oven temperature program was ramped from 40 °C to 200 °C at 10 °C min^−1^, followed by a 6 °C min^−1^ ramp to 310 °C and a 15 minutes isothermal period.

Free samples were analyzed for 32 scans at 4 cm^−1^ on a Bruker Alpha with a Bruker Platinum attenuated total internal reflection (ATR) attachment. FTIR uses a material's characteristic absorbance bands of light in the mid-infrared to identify chemical composition.

### Swelling tests

**Fig. 1:**
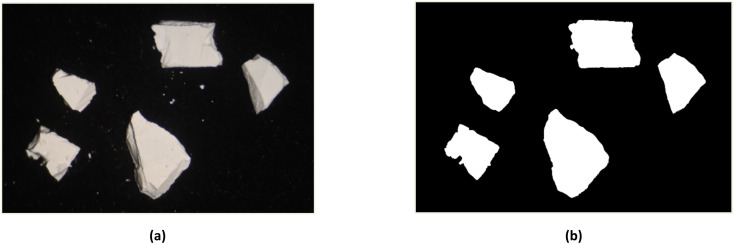
Example of frame extracted from the 15 minutes video (a) and binarized to quantify the fragment areas over time (b).

The in-plane swelling of zinc white oil paint from a 1966 painting was measured by recording size changes using a microscope and a digital camera. Images were extracted from a 15 minutes video at specific intervals and binarized to quantify the fragment areas over time (Fig. 1a and b).^11^ Visual light (VIS) images were captured using a Nikon Z 7II digital camera attached to a microscope, with a dual illuminator providing consistent lighting. Frames were extracted from the video starting at the moment when the fragments were fully saturated with solvent every twelve seconds for the first three minutes, and every forty seconds for the remaining twelve minutes. Each frame was processed in ImageJ 1.54g, converted to an 8 bit format, and binarized by adjusting the Window/Level parameters. The area of the paint fragments was measured using image thresholding. For each solvent, five to six paint fragments were analyzed. The percentage area changes for each fragment were averaged to produce a single value for each time frame, allowing for the creation of a swelling curve for each solvent. MS and ethanol (E) were used as reference solvents due to the availability of previous swelling tests in the literature, which enabled a comparison that corroborates existing findings and validates the results of this study.

## Results and discussion

### Characterization of the oil paint

**Fig. 2:**
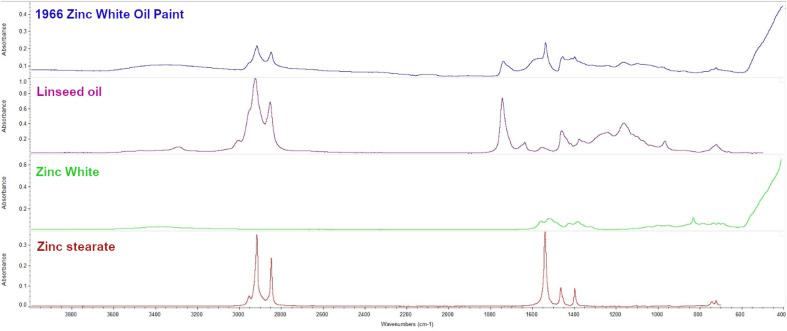
FT-IR ATR spectra: comparison between the zinc white (ZnO) oil paint, linseed oil, zinc white, and zinc stearate.

FT-IR ATR analysis confirmed that the mid-IR absorbance of the paint is consistent with a fully cured drying oil pigmented with zinc white (ZnO). A distinct band at 1536 cm^−1^ is consistent with the asymmetric stretching COO of basic zinc stearate, suggesting the paint is moderately degraded (Fig. 2).^51^

**Fig. 3:**
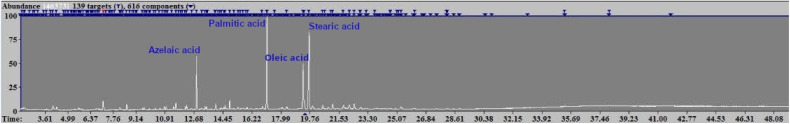
Pyrogram of the 1966 oil paint showing peaks related to azelaic, palmitic, oleic, and stearic acids.

The pyrogram of the 1966 oil paint was dominated by the methyl esters of azelaic (A), palmitic (P), oleic (O), and stearic (S) fatty acids (Fig. 3). The high A/P (0.4) and low P/S (1.3) ratios are within the typical ranges for an aged linseed oil film in a moderate state of oxidative degradation.^52^

### Swelling curves

**Fig. 4:**
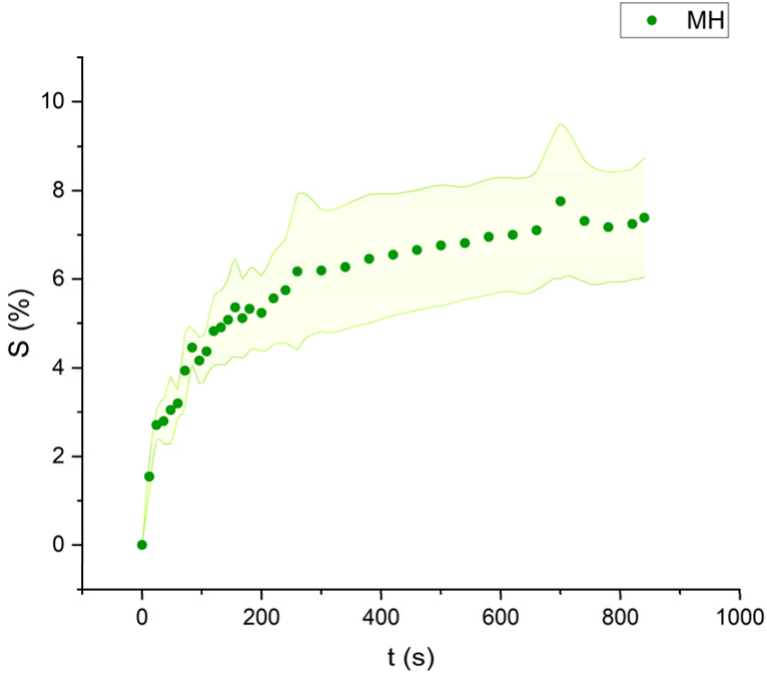
Swelling curve of Methyl Hexanoate (MH): percentage difference in surface area (*S*) over time with error band.

**Fig. 5:**
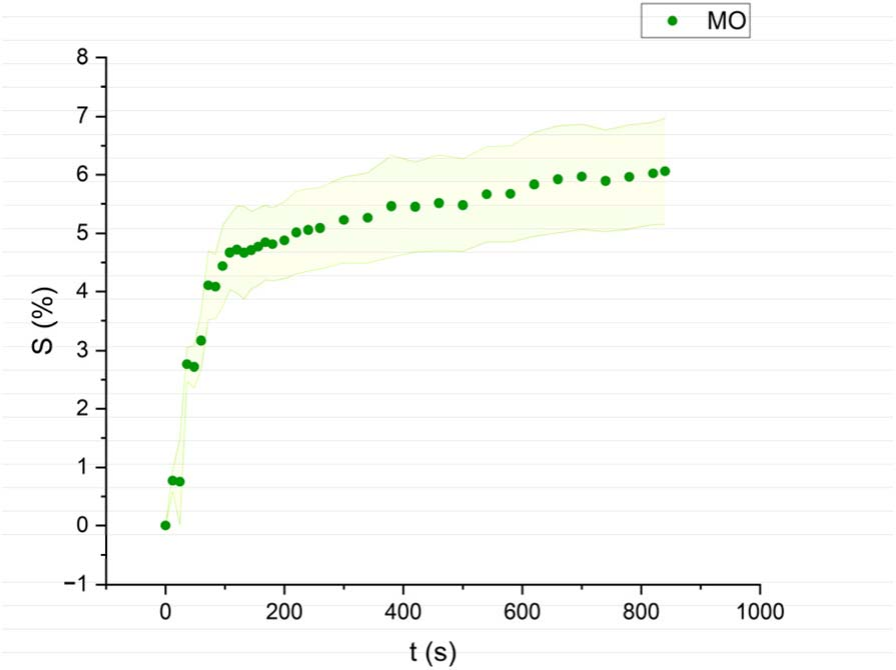
Swelling curve of Methyl Octanoate (MO): percentage difference in surface area (*S*) over time with error band.

**Fig. 6:**
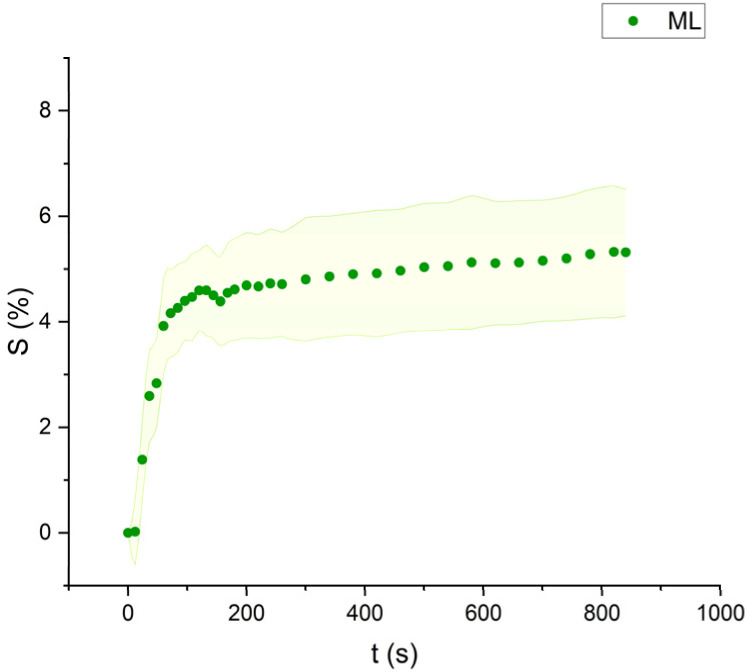
Swelling curve of Methyl Laurate (ML): percentage difference in surface area (*S*) over time with error band.

**Fig. 7:**
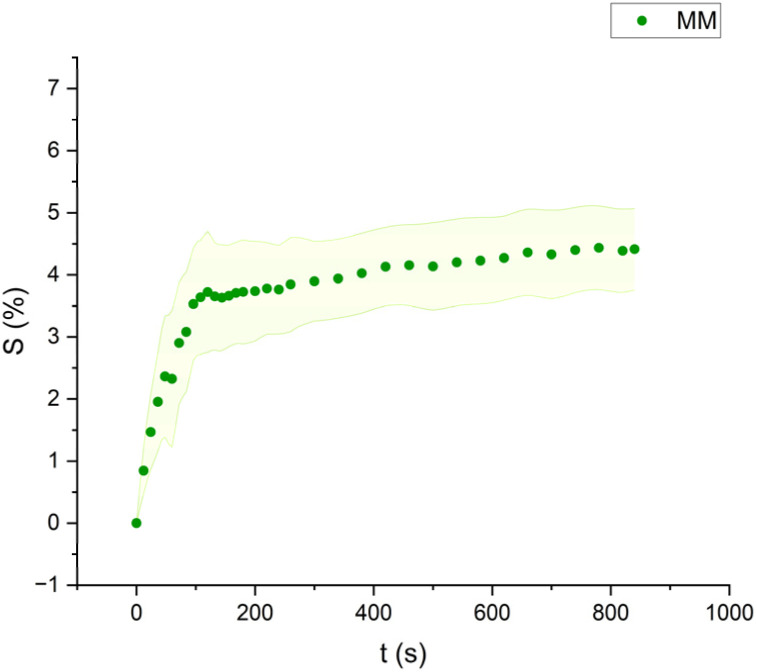
Swelling curve of Methyl Myristate (MM): percentage difference in surface area (*S*) over time with error band.

**Fig. 8:**
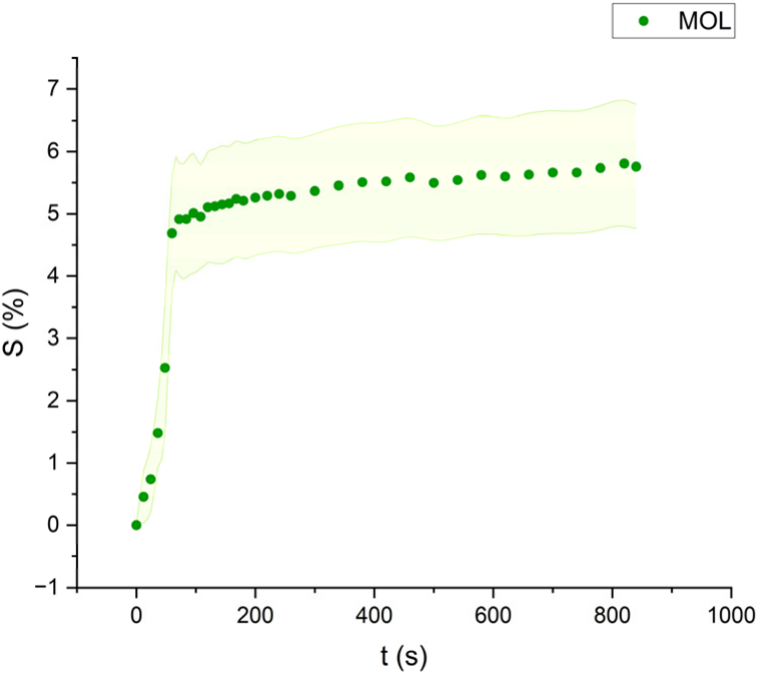
Swelling curve of Methyl Oleate (MOL): percentage difference in surface area (*S*) over time with error band.

**Fig. 9:**
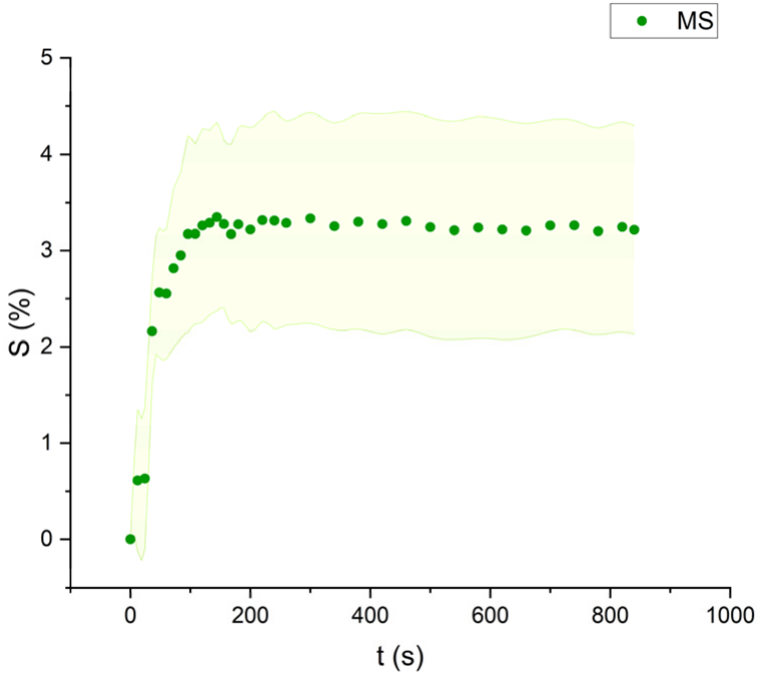
Swelling curve of Mineral Spirits (MS): percentage difference in surface area (*S*) over time with error band.

**Fig. 10:**
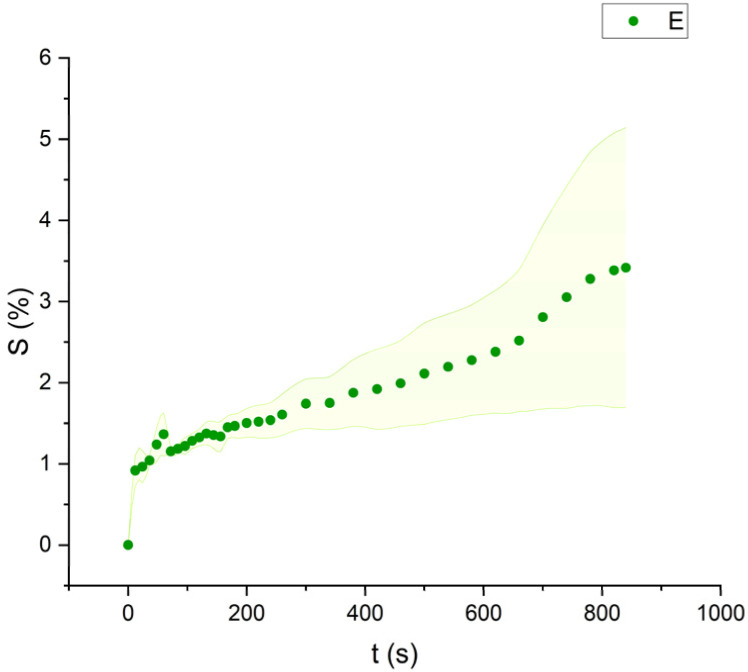
Swelling curve of Ethanol (E): percentage difference in surface area (*S*) over time with error band.

**Fig. 11:**
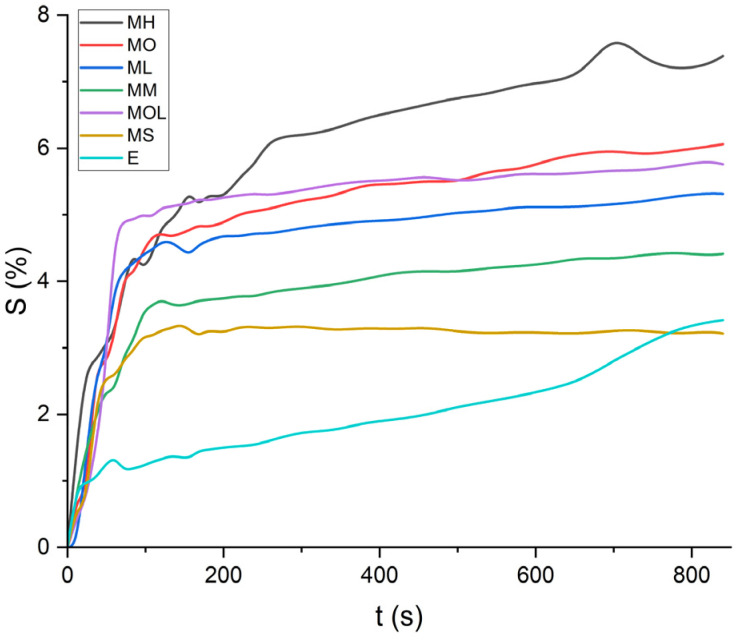
Swelling curves of the tested solvents: percentage difference in surface area (*S*) over time.

The swelling curves generated for each solvent illustrate the swelling behavior of the oil paint fragments over a 15 minutes period, showing the percentage increase in the average surface area (Fig. 4–11). The highest degree of swelling was observed with more polar solvents featuring shorter chain lengths, such as MH and MO, following a descending trend (Fig. 4, 5 and 11). As the chain length of the solvent molecules increased, the degree of swelling progressively diminished, with the exception of MOL (Fig. 6–8 and 11). The presence of a double bond in MOL's structure appears to enhance its interaction with the paint fragments, resulting in greater swelling than expected for its chain length.

Mineral spirits and ethanol exhibited low swelling capacity, with MS behaving similarly to FAMEs, while ethanol demonstrated a distinctly different pattern (Fig. 9–11). Ethanol-induced swelling was minimal (1–1.5%) in the first four minutes of contact, followed by a gradual increase that peaked at 3.4%. In contrast to MS and FAMEs, which showed a more consistent swelling trend, ethanol's swelling increased progressively over time. This difference can be attributed to the higher diffusion rate of polar solvents like ethanol and acetone, which penetrate deeper into the paint matrix and extract more LMWMs, leading to greater embrittlement compared to nonpolar solvents with the same contact time, thus making not noticeable the simultaneous swelling process.^36,44,53^ However, although the lower alcohols produced relatively low degrees of swelling in comparison with other strongly polar solvents, ethanol was shown to be appreciably more active in other studies compared to the lead white/stand oil films of Stolow.^54^

All FAME solvents demonstrated broadly similar swelling behavior, characterized by an initial rapid increase in surface area, reaching overall swelling values between 4% and 8%, identifying them as low-to-moderate swelling agents, as previously noted by Phenix (2002).^27^ However, distinct variations in swelling behavior were observed based on the polarity and chain length of the FAMEs. Shorter-chain solvents, such as MH, exhibited a more gradual swelling curve, with the most significant change occurring within the first two to three minutes, followed by a steady increase, more pronounced in MH and slightly less so in MO. In contrast, longer-chain FAMEs – ML, MM, and MOL – also displayed rapid swelling in the initial two to three minutes, but this rate slowed over time, eventually reaching a plateau.^17^ As molecular size increased, the overall extent of swelling decreased, reflected by the flattening of the swelling curve.

An interesting observation in the swelling behavior of all solvents, including the reference solvents, is a slight deflection in the curves following the initial swelling phase. This deflection, although subtle, is present to varying degrees across all solvents, with a less pronounced effect in MS, MOL, and MO. This phenomenon could be attributed to the partial release of LMWMs from the paint binder, which interact more readily with the solvent. These small molecules are easily extracted, potentially causing a slight contraction of the paint fragments – imperceptible to the naked eye. The gradual decrease and stabilization of the swelling curves may be explained by the competing processes of swelling and leaching. As the soluble and extractable components are progressively removed by the solvent, the rate of swelling slows, ultimately leading to the observed plateau.^9^

## Discussion

While the use of solvents should be minimized when possible, selecting alternative solvents with minimal impact on health and the environment is essential when their implementation cannot be avoided.^55^ To identify safer solvents for cleaning treatments on cultural heritage objects, the swelling behavior of Fatty Acid Methyl Esters (FAMEs) in naturally aged zinc white oil paints was evaluated. The observed swelling behavior of FAMEs indicates they act as low-to-moderate swellers, with variations influenced by molecular structure, particularly chain length and polarity. These findings align with earlier studies on the relationship between solvent properties and swelling, but they offer important new data specific to FAMEs, which had not been previously well-characterized in this context.

In the observed timeframe, the highest swelling was observed for the more polar FAMEs with shorter chain lengths, such as methyl hexanoate (MH) and methyl octanoate (MO), while longer-chain solvents like methyl laurate (ML) and methyl myristate (MM) induce less swelling.

**Fig. 12:**
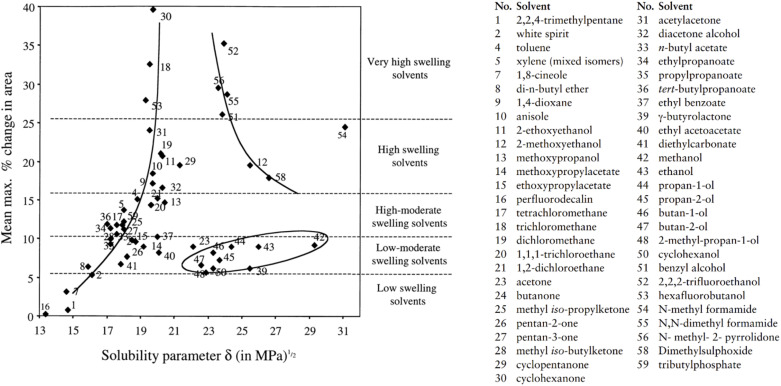
Swelling data from Phenix.^25^ Paint type – artists' oil paints containing yellow ochre and lead white pigment bound in linseed oil, not-exposed to light ageing, 140 µm thick. Maximum swelling as a function of solvent solubility parameter *∂*. *∂* values mostly from Marcus (2002).^52,56^

Despite the differences in paint properties and aging between this study and that of Phenix (2002), parallels have been found. As Phenix (2002) noted, aliphatic ester solvents generally fall within the low to moderate swelling category, producing maximum area changes of 6–12% in paint films over two hours. This aligns with the change of 4–8% within 15 minutes observed in the present study.^27^ A comparison of swelling data from Phenix's study and the maximum swelling as a function of solvent solubility parameter (*∂*) shows that the *∂* values for FAMEs, as outlined in Table 1, position them on the boundary between “low swelling solvents” and “low-moderate swelling solvents,” supporting the experimental results (Fig. 12).^27,56^

Phenix also noticed that their rates of swelling vary, essentially in relation to molecular size. This behavior aligns with well-established solvent–paint interactions, where solvents with shorter chains and higher polarity penetrate more effectively into the paint matrix, causing greater swelling. The heightened swelling seen with methyl oleate (MOL), despite its longer chain length, can be attributed to the presence of a double bond in its structure, which likely enhances interactions with the paint fragments.

It is important to note that these findings are specific to the zinc white oil paint used in this study, which is naturally aged and derived from a 1966 painting. As demonstrated in other studies, the degree of swelling and its progression over time depend significantly on the type of paint, as well as the aging of the binder and other components.^14,15^ This is particularly relevant when examining modern or contemporary paints, which may contain a variety of additives such as fillers, emulsifiers, stabilizers, or fire retardants. These additives can interact with solvents in different ways, leading to varying degrees of swelling. In addition, the composition of modern paints can also vary greatly depending on the manufacturer and the year of production.^7^

Furthermore, the results should be interpreted with consideration of the competing processes of swelling and leaching, which may affect the perceived risk for the oil paint. Indeed, an important observation was the initial rapid swelling, followed by a slight deflection and gradual stabilization across all tested solvents, including the reference ones (MS and E). This deflection may indicate a balance between swelling and leaching, where initial solvent penetration causes swelling, but as extractable molecules leach from the paint matrix, slight contraction occurs, slowing the swelling rate. This phenomenon has been documented in prior studies, suggesting that the removal of low-molecular-weight components can counterbalance the swelling caused by solvent absorption.^9,14,27,33–42^ This interplay between swelling and leaching is a critical consideration for conservators, as high-leaching solvents could extract essential components from the paint, leading to long-term damage.

## Conclusion

In conclusion, while FAME solvents show considerable potential for conservation applications, further research is required to fully understand their interactions with various types of oil paints. Specifically, the long-term impact of leaching on the stability of the paint must be thoroughly explored to ensure that FAME solvents can be used safely without compromising the integrity of the artwork.

The overall behavior of FAMEs, influenced by molecular structure, highlights the need for careful solvent selection based on the specific properties of the paint being treated. The results suggest that shorter-chain FAMEs, particularly those with higher polarity, may pose a greater risk of swelling-induced damage, while longer-chain FAMEs like MM or ML might offer a safer alternative. Nonetheless, the competing process existing between swelling and leaching phenomena must always be considered to correctly evaluate the observed change in surface area, as lower swelling may potentially correspond to greater amounts of extracted molecules such as primary and secondary plasticizers – *i.e.*, low-molecular-weight molecules.^27,29,32^ For this reason, it is important to combine swelling studies with the characterization of potentially leached material using analytical methods such as GC/MS.

Efforts to correlate swelling with single indicators—such as solubility parameters, molecular size, and vapor pressure—have often provided uncomplete results due to the complexity of paint–solvent interactions.^11–13,15,19–21,53^ Multiple factors, including the chemical composition, morphology, and aging of the treated painting, influence these interactions. As such, it is essential to consider both swelling behavior and potential leaching phenomena on a case-by-case basis, accounting for unexpected rates of swelling or contraction.

As a final point, it is crucial to highlight that, although these solvents are considered low-impact for both the environment and the operator, caution must still be exercised, and personal protective equipment should always be employed. The toxicological and ecotoxicological data available may be incomplete or subject to revision over time. Therefore, while the introduction of FAMEs in the conservation of cultural heritage marks a significant step toward a more sustainable approach, conservators should consistently refer to the latest toxicological information from recognized authorities, such as the European Chemicals Agency (ECHA) and the Environmental Protection Agency (EPA).

## Data availability

The data supporting this article have been included as part of the ESI.†

## Author contributions

Conceptualization, C. B. and M. D.; methodology, C. B. and M. D.; validation, C. B. and M. D.; formal analysis, C. B. and M. D.; investigation, C. B. and M. D.; resources, M. D.; data curation, C. B. and M. D.; writing – original draft preparation, C. B.; writing – review and editing, C. B., M. D. and G. F.; visualization, C. B.; supervision, M. D. and G. F.; project administration, M. D. and G. F.

## Conflicts of interest

There are no conflicts to declare.

## Supplementary Material

RA-014-D4RA07464E-s001
