# Supplementary material for: Assessing the swelling behavior of oil paint in fatty acid methyl esters (FAMEs)
Source: RSC Adv. 2024 Dec 17;14(53):39692–9. doi: 10.1039/d4ra07464e (PMC11650782; doi:10.1039/d4ra07464e)
Supplement: RA-014-D4RA07464E-s001 [file RA-014-D4RA07464E-s001.pdf]

Supplementary Information (SI) for RSC Advances.
This journal is © The Royal Society of Chemistry 2024

Time (s)	Methyl Hexanoate		Methyl Octanoate		Methyl Laurate		Methyl Myristate		Methyl Oleate		Mineral Spirits		Ethanol	
	Swelling (%)	Error	Swelling (%)	Error	Swelling (%)	Error	Swelling (%)	Error	Swelling (%)	Error	Swelling (%)	Error	Swelling (%)	Error
0	0	0	0	0	0	0	0	0	0	0	0	0	0	0
12	1.545	0.34035	0.76681	0.17852	0.02184	0.62747	0.84444	0.37304	0.45441	0.42148	0.6134	0.73873	0.91829	0.18977
24	2.71072	0.33368	0.75082	0.73475	1.38646	0.75302	1.46947	0.60687	0.73932	0.52859	0.63265	0.73616	0.96364	0.19915
36	2.79628	0.51221	2.76171	0.28851	2.59118	0.87142	1.95499	0.79266	1.48406	0.55511	2.16045	0.54658	1.04139	0.06137
48	3.04845	0.75752	2.71941	0.36344	2.83412	0.84329	2.36244	0.97507	2.5285	1.0693	2.56409	0.66921	1.239	0.21249
60	3.19855	0.32066	3.16256	0.48342	3.92046	0.89913	2.3261	1.1012	4.68831	0.92578	2.55325	0.67238	1.36468	0.26541
72	3.93786	0.83919	4.10849	0.58764	4.16182	0.83223	2.9017	0.9805	4.91253	0.90272	2.81577	0.8234	1.15333	0.0411
84	4.46173	0.40325	4.08539	0.55567	4.26239	0.83291	3.07982	0.96498	4.9162	0.94386	2.95047	0.86415	1.18486	0.04596
96	4.16572	0.51764	4.43535	0.68945	4.39599	0.73625	3.53109	0.90332	5.00889	0.96657	3.17218	1.02029	1.22088	0.10618
108	4.37202	0.53377	4.66732	0.63577	4.4672	0.82694	3.63794	0.92035	4.95347	0.82777	3.17441	0.93703	1.28253	0.09212
120	4.8212	0.7746	4.71926	0.74584	4.59268	0.7512	3.72383	0.97653	5.10647	0.88875	3.26162	1.00635	1.32426	0.10326
132	4.90279	0.83289	4.66483	0.79646	4.59674	0.86053	3.65308	0.86457	5.12	0.92096	3.28972	0.96005	1.37459	0.14129
144	5.07745	0.94187	4.71039	0.66295	4.49897	0.82151	3.63187	0.85295	5.15	0.95036	3.35026	0.98166	1.35533	0.16614
156	5.35979	1.1089	4.76703	0.66142	4.3866	0.84252	3.66322	0.81771	5.16566	0.91692	3.27706	0.87263	1.33777	0.18604
168	5.11331	0.89002	4.84264	0.63916	4.54891	0.93799	3.70852	0.81592	5.23782	0.93505	3.17143	0.93662	1.45092	0.13888
180	5.32425	0.92709	4.81177	0.62334	4.61316	0.96423	3.72464	0.83684	5.21	0.92902	3.27405	1.00175	1.46645	0.14614
200	5.23342	0.84773	4.87678	0.65565	4.6889	0.99703	3.73781	0.80235	5.26	0.92902	3.21958	1.06144	1.50388	0.17726
220	5.56317	1.0366	5.01171	0.70948	4.668	0.98441	3.78143	0.73825	5.29055	0.92123	3.3174	1.05598	1.52036	0.20352
240	5.74385	1.16023	5.05544	0.7079	4.72535	1.03129	3.7648	0.71226	5.32	0.92403	3.31281	1.12977	1.53879	0.21691
260	6.17	1.76422	5.08741	0.70136	4.71112	0.98632	3.84492	0.7588	5.29	0.92139	3.28742	1.05856	1.60597	0.25503
300	6.18992	1.37546	5.22551	0.73089	4.80262	1.1711	3.89665	0.64115	5.36552	0.91824	3.33613	1.09556	1.74146	0.30564
340	6.26944	1.40747	5.26337	0.77248	4.8566	1.14519	3.94031	0.63734	5.45393	0.94694	3.2538	1.07414	1.7492	0.32507
380	6.45496	1.45903	5.46386	0.86635	4.90196	1.15058	4.0261	0.64349	5.50784	0.9497	3.29971	1.11942	1.87492	0.41119
420	6.5484	1.3776	5.4516	0.77355	4.91544	1.19868	4.13289	0.63125	5.51939	0.9661	3.27576	1.14552	1.92054	0.49392
460	6.65522	1.35735	5.51754	0.82226	4.96604	1.16258	4.15636	0.65106	5.586	0.95551	3.30714	1.13015	1.9928	0.52607
500	6.7591	1.36942	5.48002	0.79506	5.03567	1.20395	4.13772	0.706	5.49803	0.91962	3.24343	1.14177	2.1122	0.62503
540	6.81121	1.27107	5.66591	0.81678	5.05316	1.20256	4.20219	0.70036	5.54224	0.92333	3.21085	1.13394	2.19455	0.65144
580	6.95143	1.31044	5.67355	0.82365	5.12488	1.26657	4.22748	0.69714	5.62251	0.95112	3.23951	1.1533	2.27846	0.67889
620	6.9983	1.2894	5.83545	0.88649	5.1114	1.16914	4.27085	0.67686	5.59958	0.94074	3.21936	1.14598	2.38104	0.75649
660	7.10102	1.33496	5.92419	0.91363	5.12223	1.17294	4.36093	0.69362	5.63046	0.9799	3.20896	1.11265	2.51944	0.8708
700	7.7599	1.75832	5.96622	0.90123	5.15649	1.1457	4.32984	0.71247	5.66535	0.9858	3.26096	1.09918	2.80734	1.13331
740	7.312	1.36929	5.89676	0.86714	5.19984	1.17587	4.39952	0.68387	5.66348	0.97651	3.26278	1.09044	3.05255	1.36834
780	7.17328	1.24531	5.96223	0.89128	5.28169	1.22507	4.43523	0.67215	5.73677	0.99655	3.20176	1.07508	3.27969	1.56492
820	7.24296	1.25146	6.022	0.8776	5.32236	1.25085	4.38732	0.67042	5.80802	1.01053	3.24515	1.09192	3.38413	1.69297
840	7.38573	1.35143	6.06023	0.91055	5.314	1.19936	4.41234	0.65516	5.7591	0.99606	3.21546	1.084	3.41614	1.72569
